# EP16 Challenges in treating new onset systemic lupus erythematosus with lupus nephritis and COVID-19 infection overlap

**DOI:** 10.1093/rap/rkaa052.015

**Published:** 2020-11-03

**Authors:** Selma Dahou, Tim Leach, Kathryn Bostock, Jonathan Louden, Jonathan Raj, Shivan Ghedia

**Affiliations:** r1 Queen Alexandra University Hospital, Portsmouth, United Kingdom; r2 Wessex Kidney Centre, Portsmouth, United Kingdom

## Abstract

**Case report - Introduction:**

COVID-19 infection caused by a novel coronavirus SARS-coV-2 has made the diagnosis and the treatment of inflammatory diseases incredibly challenging. On the one hand, because of its pro-inflammatory state, that may aggravate or trigger flares in autoimmune diseases such as systemic lupus erythematosus (SLE). On the other hand, the risk of an immunosuppressive therapy during the active phase SARS-coV-2 infection that may lead to catastrophic outcomes. We report a case of a 24-year-old female newly diagnosed with SLE during COVID-19 pandemic who developed COVID-19 infection during her induction treatment for lupus nephritis.

**Case report - Case description:**

A 24-year-old Nepali female, with no past medical history of note, presented to her regional hospital with a history of flu-like symptoms few days ago, peripheral oedema, acute kidney injury with proteinuria and hypertension. Further investigations showed a high titre of double-stranded DNA antibodies, anti-cardiolipin IgM and B2 microglobulin positive and low C3. She also developed a haemolytic anaemia and thrombocytopenia during her admission. She received pulsed steroid therapy and was started on mycophenolate mofetil (MMF) for a probable lupus nephritis awaiting the results of biopsy, which showed later a lupus nephritis Class IV-G with active lesions. She then developed symptoms of COVID-19 infection and had a positive PCR leading to an interruption of her induction therapy. She was recruited to the RECOVERY trial on the lopinavir-ritonavir arm and made a good recovery.

**Case report - Discussion:**

It is well known that viruses can trigger or aggravate auto-immune response in patients predisposed genetically. However, the role of SARS-coV-2 is not elucidated yet. The EULAR COVID-19 registry showed that rheumatoid arthritis and SLE were the most prevalent rheumatic diseases, and there was an increased risk in those who are on moderate to high dose corticosteroids. In patients with SLE and COVID-19 infection, it is agreed by all the national and international rheumatology societies to interrupt their immunosuppressive therapy until the symptoms resolve, especially those with renal involvement or an active disease. Which is the case in our patient. Luckily, she resumed her MMF a month later after a negative PCR and her renal function has continued to improve.

**Case report - Key learning points:**

Lupus nephritis is a major risk factor for overall morbidity and mortality in SLE. It requires an early immunosuppressive treatment to induce remission.

Randomized clinical trials showed that MMF is at least equally effective as cyclophosphamide in inducing remission and that it has been associated with a reduced risk of infection and amenorrhea. It seems to be a suitable alternative in women of childbearing age. In patients with concomitant COVID-19 disease, immunosuppressive therapy should be paused until the symptoms improve.

